# Assessment of Heavy Metal Pollution and Potential Ecological Risk in Sewage Sludge from Municipal Wastewater Treatment Plant Located in the Most Industrialized Region in Poland—Case Study

**DOI:** 10.3390/ijerph16132430

**Published:** 2019-07-09

**Authors:** Malwina Tytła

**Affiliations:** Institute of Environmental Engineering, Polish Academy of Sciences, 34 M. Skłodowskiej-Curie St., 41-819 Zabrze, Poland; malwina.tytla@ipis.zabrze.pl

**Keywords:** heavy metal pollution, ecological risk assessment, chemical sequential extraction, sewage sludge, wastewater treatment plant, Silesian Voivodeship

## Abstract

This study aimed to assess the pollution and potential ecological risk of seven heavy metals (Cd, Cr, Cu, Hg, Ni, Pb, and Zn) in the sewage sludge collected from a wastewater treatment plant (WWTP), located in the most industrialized region of Poland (Silesian Voivodeship). The concentrations of heavy metals were determined using inductively coupled plasma optical spectrometry (ICP-OES) and cold vapor atomic absorption spectrometry (CVAAS). The chemical forms (chemical speciation) of heavy metals were determined using the three-step chemical sequential extraction procedure, developed by the Community Bureau of Reference (BCR). To assess the pollution level and potential ecological risk, the following indices were used: Geoaccumulation Index (I_geo_), Potential Ecological Risk Factor (ER), Individual Contamination Factor (ICF), modified Risk Assessment Code (RAC_m_), and Ecological Risk Factor (ERF)—the author’s index. Sludge samples were collected at successive stages of processing. The results revealed that the activated sludge process and sludge thickening have a significant impact on heavy metal distribution, while anaerobic digestion and dehydration decrease their mobility. The most dominant metals in the sludge samples were Zn and Cu. However, the content of heavy metals in sewage sludge did not exceed the permissible standards for agricultural purposes. The concentrations of heavy metals bound to the immobile fractions exhibited higher concentrations, compared to those bound to mobile fractions (except Zn). The values of the total indices indicated that sludge samples were moderately to highly contaminated with Zn, Hg, Cd, Cu, and Pb, of which only Hg, Cd, and Cu posed a potential ecological risk, while according to the speciation indices, sludge samples were moderately to very highly polluted with Zn, Cu, Cd, Cr, and Ni, of which Zn, Ni, and Cd were environmentally hazardous. The obtained results proved that assessment of the pollution level and potential ecological risk of heavy metals in sewage sludge requires knowledge on both their total concentrations and their chemical forms. Such an approach will help prevent secondary pollution of soils with heavy metals, which may influence the reduction of health risks associated with the consumption of plants characterized by a high metal content.

## 1. Introduction

Sewage sludge is a waste organic material generated in wastewater treatment plants (WWTPs), as a by-product of wastewater treatment [1,2]. Therefore, the final quality of sludge mainly depends on the chemical composition of raw wastewater and processes used in the WWTPs. The production of sewage sludge increases every year, which results from population growth and the increasing effectiveness of biological wastewater treatment processes [3,4,5]. It is indicated in the literature that by 2020 the total production of sewage sludge in European Union (EU) countries will reach 12,997,000 tons of dry matter (DM), of which 950,000 tons will be generated in Poland (Central Europe) [6]. 

Sewage sludge is composed of high concentrations of organic matter (OM) and biogenic compounds, especially nitrogen (N) and phosphorus (P), which are necessary for plant growth [1,2,7]. However, it also contains heavy metals, including those classified as toxic, i.e., cadmium (Cd), chromium (Cr), copper (Cu), mercury (Hg), nickel (Ni), lead (Pb) and zinc (Zn) [8,9]. This means that, depending on the concentration and exposure time, a given metal can pose both environmental and health risks, which is associated with its ability to bioaccumulate in the food chain [10]. The most common sources of heavy metals in sewage sludge are domestic and industrial wastewaters and corrosion of sewerage systems, as well as surface runoff from urbanized areas or roads [11,12]. WWTPs may also receive wastewater from the agro-industrial sector. Other sources of heavy metals are pharmaceuticals, as well asbody care and cleaning products. Moreover, illegal wastewater discharges are also an important source of sewage sludge pollution with heavy metals [13]. 

Heavy metals present in the influent wastewater accumulate in sewage sludge (approximately 80–90%), which may affect its treatment and final characteristics [14]. Unfortunately, processes commonly used in WWTPs do not definitely guarantee the removal of heavy metals, which in turn can lead to secondary environmental pollution [10]. Therefore, the effective protection of the natural environment against pollution from sewage sludge is of great concern nowadays. Even more important, is having regard for the fact that the most preferable method for sludge management is its use as a fertilizer or substrate for soil remediation [3,8]. In the EU countries, from 30% to 50% of sewage sludge is used in agriculture, which constitutes an alternative to landfill or incineration [4,7]. In Poland, one of the most important criteria for agricultural use of sludge is heavy metal concentrations; for which limits are regulated by the Regulation of the Minister of Environment of 6th February 2015 on municipal sewage sludge (J. L. 2015, item. 257) [15], being compatible with the Council Directive of 12 June 1986 on the protection of the environment, and in particular of the soil, when sewage sludge is used in agriculture (86/278/EEC) [16]. It is worth noting that high concentrations of heavy metals in sewage sludge may cause contamination of soil, and surface and ground water as well as crops, and as a consequence, have a negative impact on living organisms, including animals and humans [3]. However, knowledge of the total concentrations of heavy metals allows only for the assessment of the degree of sludge pollution and is inconclusive regarding the potential ecological risk that these elements may pose to the environment and living organisms. This is due to the fact that the mobility, bioavailability, and toxicity of heavy metals depend on their speciation forms (in this case the chemical), which are influenced by their leaching and interactions with different components of natural ecosystems [2,17,18]. The most commonly used method to determine the chemical forms of heavy metals in sewage sludge, is the three-step chemical sequential extraction procedure proposed by the Community Bureau of Reference (BCR; now the Standards, Measurements and Testing Programme) [17,19,20,21], which is a modification of the Tessier method [22]. It is worth mentioning that every change in environmental conditions, such as pH value, redox potential (Eh), organic matter content (OM), etc., results in changes to the mobility of metals and thereby, in their bioavailability [4]. Moreover, heavy metals bound to the sludge structure, can also undergo transformation while being subjected to the biological, chemical, and mechanical processes used in WWTPs [8]. Therefore, it is important to analyze not only total concentrations but also chemical forms, during various stages of sludge processing. This approach results in more precise assessment of the potential ecological risk posed by these elements. So far, there are not many publications in this research area in the scientific literature and these works are mostly devoted to one selected type of sludge, i.e., dewatered [17,21]. However, the approach presented in this study allows a more comprehensive analysis of the ecological risk to be conducted, which includes sludge characteristics for the whole technological line of the WWTP.

A variety of methods have been proposed in order to assess the pollution level and ecological risk of heavy metals in sewage sludge. In this study two different types of indices were used, i.e., total content indices and speciation indices [23]. The first group, total content, comprised the Geoaccumulation Index (I_geo_) [24] and Potential Ecological Risk Factor (ER) [25], whereas the second group, speciation indices, comprised the Individual Contamination Factor (ICF) [23,26], modified Risk Assessment Code (RAC_m_) (based on criteria specified in reference) [27], and Ecological Risk Factor (ERF)—the author’s index. These indices are calculated in different ways and include various aspects. For example, I_geo_ considers the accumulation level of heavy metals in sewage sludge without toxicity impact; ER focuses both on the total quantity of heavy metals and toxicity, while ICF, RAC_m_, and ERF focus on heavy metal mobility [11]. In previous years, the above-mentioned indices have been successfully used to assess the ecological risk in soils and sediments [28,29]. 

The Silesian Voivodeship is one of the most urbanized areas in Poland and the largest industrial region in the country. The diversity of the industrial sector in this region, e.g. metallurgical coal, mining, energy production, heavy industry, and transportation, contributes to the environmental pollution with heavy metals [30,31], which in turn, has an impact on the chemical composition of wastewater discharged into WWTPs, and as a consequence, on the characteristics of sewage sludge. In 2017, the production of sewage sludge in the Silesian Voivodeship reached 64,039 tons of DM, of which 10,317 tons were used for agricultural purposes [32]. Thus, both the geographical location of the studied WWTP, as well as the presence of heavy industry and strong environmental pollution by heavy metals in this area, provided legitimacy for carrying out this research.

The aims of this study were: (1) to determine the total concentrations of heavy metals (Cd, Cr, Cu, Hg, Ni, Pb, and Zn) in sewage sludge at different processing stages; (2) to investigate the chemical forms of heavy metals in sewage sludge by using the BCR sequential extraction procedure; and (3) to assess the pollution level and potential ecological risk of heavy metals in sewage sludge.

## 2. Materials and Methods

### 2.1. Study Area and Sampling

**Figure 1:**
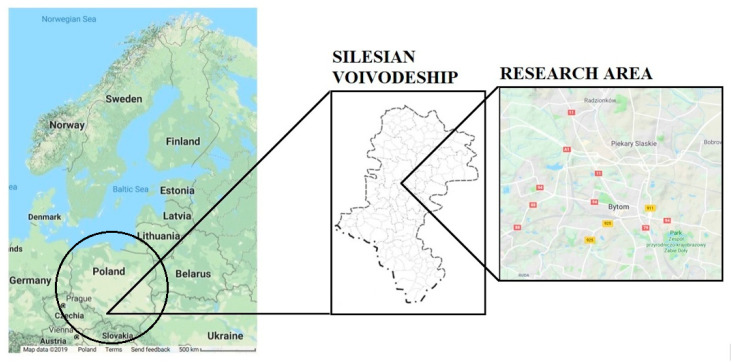
The maps present the location of study area. Original source files from: https://www.google.pl/maps/@52.0122001,29.5346949,3.17z/data=!5m1!1e4?hl=en; https://www.google.pl/maps/@50.3650717,18.80662,12z/data=!5m1!1e4?hl=enfgg [33,34].

**Figure 2:**
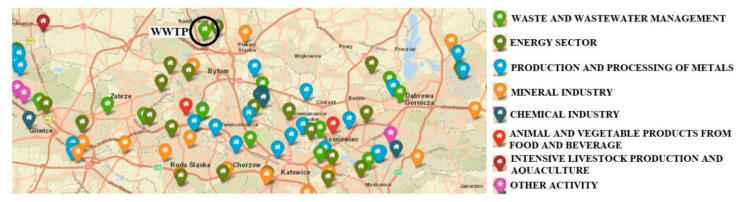
Industrial activity in the Silesian Voivodeship. The map presents a fragment of the largest industrial region in Poland (with “Central” wastewater treatment plant (WWTP) in Bytom marked). Original source files from: https://prtr.eea.europa.eu/#/industrialactivity [35].

The sewage sludge samples were collected in October 2018 from the municipal “Central” wastewater treatment plant in Bytom, Silesian Voivodeship, Southern Poland (Central Europe). The WWTP is located in the most important industrial region in Poland (Figure 1). Examples of industrial activities in the Silesian Voivodeship are shown in Figure 2. 

Sludge samples were collected twice, at 1-hour intervals, at seven sampling points constituting stages in the technological line of the WWTP: primary sludge—from settling tank (S1), primary sludge—after thickening (S2), secondary sludge—from settling tank/after activated sludge process (S3), secondary sludge—after thickening with addition of conditioning agent (S4), mixed sludge—from sampling point numbers 2 and 4 (S5), digested sludge—from anaerobic digester (S6), and dewatered sludge from belt press (S7). This approach was intended to capture changes in the physicochemical characteristic of sludge at different stages of processing, and to obtain the most representative samples. All samples were kept in polypropylene containers and stored in a refrigerator at 4 °C, until analysis. The operational parameters of the WWTP are shown in Table 1.

### 2.2. Physicochemical Analysis

#### 2.2.1. Measurement of pH, Redox Potential (Eh), Dry Matter (DM) and Organic Matter (OM)

The pH and Eh of sewage sludge were measured using a multifunctional meter CPR-411 (Elmetron) and electrodes as in order: IJ44A (Elmetron) and ERS-2 (Elmetron). The content of DM and OM in sewage sludge was determined after drying at 105 °C and then ignition at 550 °C, according to Polish Standards, i.e., characteristics of sewage sludge, determination of dry residue and water content (PN-EN 12880:2004) [36] and characteristics of sewage sludge, determination of loss on ignition of dry matter (PN-EN 12879:2004) [37]. 

#### 2.2.2. Determination of Total Heavy Metal Concentrations

Determination of the total heavy metal concentrations (Cd, Cr, Cu, Hg, Ni, Pb, and Zn) included sludge sample preparation, i.e., initial drying for 48 h at room temperature until air-dried and then to constant mass at 105 °C. In the next step, sludge samples were milled in a mortar grinder. Afterwards, 0.2 g of sample was digested with nitric acid (HNO_3_) and hydrochloric acid (HCl) in a Teflon flask, using a microwave digestion system (Multiwave 3000, Anton Paar GmbH, Graz, Austria). Obtained solutions were filtered through fine filters (0.45 µm) and diluted with 5% HNO_3_ to a volume of 50 ml. Samples were stored at 4 °C prior to analysis. The total heavy metal concentrations were determined using inductively coupled plasma optical spectrometry (Avio 200 ICP-OES, PerkinElmer Inc., Waltham, MA, USA). Mercury was assayed with cold vapor atomic absorption spectrometry (CVAAS). All standards were prepared on the day of analysis. The limits of detection (LODs) were 0.4, 0.4, 0.4, 0.1, 0.8, 0.8, and 4.2 mg kg^−1^ for Cd, Cr, Cu, Hg, Ni, Pb, and Zn, respectively. The measurements were performed in triplicate.

#### 2.2.3. Sequential Extraction of Heavy Metals in Sewage Sludge

To determine the chemical forms of heavy metals in sludge samples, the three-step chemical sequential extraction procedure of the Community Bureau of Reference (BCR; now the Standards, Measurements and Testing Programme), was used. Additionally, after completion of the sequential extraction procedure, the residual heavy metal contents were determined. The concentrations of heavy metals in water extracts were analyzed with inductively coupled plasma optical spectrometry (Avio 200 ICP-OES, PerkinElmer Inc., Waltham, MA, USA). The experiments were performed in triplicate. A scheme of the sequential extraction is shown in Table 2.

### 2.3. Pollution Level and Ecological Risk

To assess the pollution level and potential ecological risk of heavy metals in sewage sludge, the following indices were used: Geoaccumulation Index (I_geo_) [24], Potential Ecological Risk Factor (ER) [25], Individual Contamination Factor (ICF) [23,26], modified Risk Assessment Code (RAC_m_) (based on criteria specified in reference [27]) and Ecological Risk Factor (ERF)—the author’s index. The I_geo_ and ER indices refer to total concentrations of heavy metals, while ICF, RAC_m_, and ERF refer to their chemical forms. The pollution levels and ecological risks of heavy metals in sewage sludge were calculated by the equations shown in Table 3. However, the observations made so far have shown that some metals are characterized by a low or moderately low percentage share in the most mobile fraction F1, such as Cu or Zn, or they do not show this share at all, such as Cd, and yet still by their share in fraction F2 indicate the existence of a potential threat to the environment (confirmed by the other ecological risk indices). In view of the above, it was decided to modify the original formula for calculating the RAC index. The original formula took into account the percentage share of a single metal only in the first fraction (F1). In turn, the proposed modification consisted in taking into account the share of a given metal also in the second fraction (F2). The category and description have remained unchanged.

### 2.4. Statistical Analysis

All calculations were performed using Statistica 12.0 (StatSoft) and Excel 2013 (Microsoft Office Standard). The occurrence of a linear correlation between analyzed variables was evaluated by Pearson’s correlation coefficient (r). Data analysis also included means ( $\bar{x})$ and standard deviations (SD).

## 3. Results and Discussion

### 3.1. Physicochemical Characteristics of Sewage Sludge

The physicochemical characteristics of sewage sludge are shown in Table 4. The pH of sludge samples ranged from 5.4 to 7.4. The lowest pH levels were found in the thickened primary sludge (S2) and mixed sludge (S5). Moreover, the redox potential was in the range of −350.0 to −175.0 mV. The lowest Eh was found for thickened primary sludge (S2), while the highest was found for primary sludge (S1). Furthermore, the sludge samples at particular sampling points differed in their dry matter content. It is likely that this was related to the type of treatment that was applied to the sludge processing. The dry matter content ranged from 0.6% to 20.3% for primary and dehydrated sludge, respectively, which also reflected the level of sludge hydration, i.e., 99.4% and 79.7%, respectively. The highest organic matter content was found in the primary sludge (83.3%_DM_), while the lowest was found for sludge after anaerobic digestion (61.8%_DM_). The above results are consistent with those presented in other studies [8,39].

### 3.2. Total Heavy Metal Concentrations

The total heavy metal concentrations in sludge samples are shown in Table 5. The experiment conducted showed that the mean concentrations of heavy metals in sewage sludge at different stages of processing, were in the following order: Zn > Cu > Pb > Cr > Ni > Cd > Hg (S1); Zn > Cu > Pb > Ni > Cr > Cd > Hg (S2, S3, S7); Zn > Pb > Cu > Ni > Cr > Cd > Hg (S5, S6). Slight fluctuations were observed with regard to the order of individual metals (Cu–Pb, Cr–Ni). However, Zn and Cu were found to be the most dominant heavy metals in sewage sludge at different stages of processing, while Cd and Hg were the least noticeable. The above observations are in good agreement with the results obtained by other researchers [2,20,40]. Moreover, it should be emphasized that according to the literature, Cd and Hg (in excess concentrations) are highly toxic, mainly to humans and animals (and are less toxic to plants), while Zn and Cu inversely [41]. This is even more important when taking into account that the main method of sludge management is its agricultural use. However, in this study, the total concentrations of heavy metals did not exceed the permissible standards for sewage sludge in Poland (J. L. 2015, item. 257) [15] and EU (86/278/EEC) [16].

The lowest content of heavy metals was found in the thickened primary sludge and mixed sludge, i.e., 1382.1 mg·kg^−1^ and 1764.4 mg·kg^−1^, respectively, whereas the highest was found in sewage sludge after anaerobic digestion and dehydration, i.e., 2400.8 mg·kg^−1^ and 2396.7 mg·kg^−1^, respectively. According to Álvarez et al. (2002) [20], this was probably related to the weight loss of the fresh sludge during the anaerobic digestion process, and at the later stage to an increase of dry matter content during sludge dehydration. The impact of an increase in DM content on the total heavy metal concentrations in sludge was also observed in the case of dewatered sludge, which was characterized with higher DM and metal contents compared to primary or secondary sludge. The only exception was primary and secondary sludge after thickening, in which, despite the increase in dry matter content, the total metal content decreased compared to the non-thickened sludge, which in turn affected the characteristics of the mixed sludge. It can be assumed that as a result of the thickening processes, the heavy metals in the sewage sludge have probably passed into the liquid phase. Another reason for this phenomenon may also be poor mixing of the sludge and the conditioning agent (in the case of secondary sludge). In addition, it was also observed that in the sludge after anaerobic digestion, despite the decrease in dry matter content, the total heavy metal concentrations were maintained at high levels. This could be related to the good binding of these elements to the solid particles of sludge or to the changes in its characteristics during the anaerobic digestion. The above results partly confirm the author's previous observations [8,42].

Table 6 presents the results of the Pearson’s correlation analysis for heavy metals in sewage sludge. The obtained results indicate the presence of strong positive correlations between the selected elements, with the exception of Hg. Similar observations have also been made by other researchers [3,21,40]. It can be assumed that statistically significant correlations among the heavy metals may prove that they possibly have a similar accumulation behavior or originate from the same sources of pollution. However, in this case, further studies and analysis should be conducted.

### 3.3. Chemical Speciation of Heavy Metals

**Figure 3:**
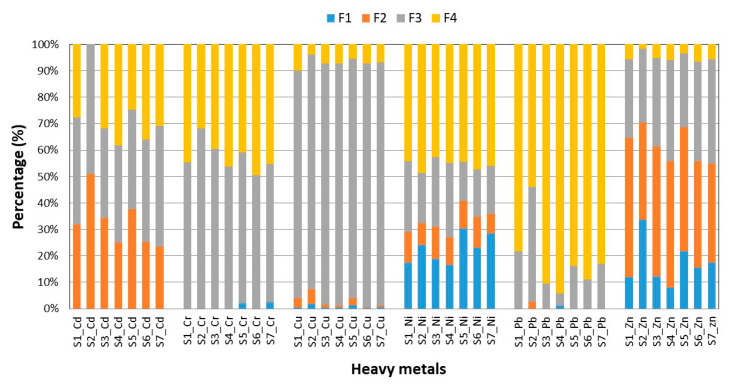
Heavy metal distribution in sewage sludge.

The results of BCR sequential extraction of heavy metals in the sludge samples are shown in Table 7, whereas the distribution of heavy metals is presented in Figure 3. The sequential extraction analysis did not cover Hg, due to its very low concentration in the sludge samples. Moreover, in some cases the concentrations of Cd, Cr and Pb bound to different chemical forms, were below their limits of detection (LODs).

Verification of the BCR sequential extraction procedure was performed by comparing the sum of the four fractions with the total concentrations of heavy metals in sludge samples, i.e., using recovery rate (R). The recovery rate of heavy metals ranged from 41.1% to 124.4% (Table 7). The obtained results confirmed that the BCR sequential extraction procedure is adequate and reliable for detecting the speciation of analyzed heavy metals. Similar results were obtained by other researchers, for example, 64.5–106.0% [43], 29.7–117.9% [8], and 81.2–130.9% [21]. The research conducted revealed that Zn and Cu occurred in the largest quantities, i.e., 14.3–600.5 mg·kg^−1^ and 0.5–210.5 mg·kg^−1^, respectively, while Cd occurred in the smallest quantities, i.e., 0.7–2.2 mg·kg^−1^ (Table 7).

The distribution of individual heavy metals in the selected fractions of sewage sludge varied at particular sampling points: Cd—F3 > F4 > F2> F1 (S1), F2, F3 > F1, F4 (S2), F2,F3 > F4 > F1 (S3,S5), F4 > F3 > F2 > F1 (S4), F3 > F4 > F2 > F1 (S6,S7); Cr – F3 > F4 > F1, F2 (S1,S2,S3,S4,S6), F3 > F4 > F1 > F2 (S5,S7); Cu—F3 > F4 > F2 > F1 (S1-S7); Ni - F4 > F3 > F1 > F2 (S1,S3,S4), F4 > F1 > F3 > F2 (S2,S5,S6,S7); Pb—F4 > F3 > F1,F2 (S1), S4 > F3 > F2 > F1 (S2); F4 > F3 > F1 > F2 (S4), F4 > F3 > F1,F2 (S3,S5,S6,S7) and Zn—F2 > F3 > F1 > F4 (S1,S3,S4,S5,S6), F2 > F1 > F3 > F4 (S2,S7). In summary, sludge at different stages of its processing exhibited higher concentrations of heavy metals bound to the immobile fractions (F3 and F4), compared to the mobile ones (F1 and F2). The observations presented above were confirmed by other researchers [8,17,20]. The exception was zinc, in which case the percentage share in the mobile fractions prevailed over the mobile ones and ranged from 55.0% (S7) to 70.4% (S2). Also noteworthy are cadmium and nickel, for which the share in the mobile fractions ranged from 23.4% (S7) to 51.0% (S2), and from 27.1% (S4) to 40.8% (S5), respectively. Similar results were presented by Yang et al. (2017) [17] who indicated that the percentage share of Zn, Cd, and Ni in the mobile fractions of sewage sludge ranged between 59.57% and 79.12%, 48.90% and 60.52%, and between 34.67% and 70.34%, respectively. Also, other scientists confirmed that zinc showed the greatest bioavailability among the heavy metals considered [20,21,40]. The obtained results also revealed that chromium, copper, and lead were predominantly present in the immobile fractions, with percentage shares in the ranges 97.6 (S7)–99.5% (S2); 92.7 (S2)–99.4% (S6) and 97.3 (S2)–98.8% (S4), respectively (Figure 3). Generally, in this study, the distribution of heavy metals bound to the mobile fractions decreased gradually over successive stages of sewage sludge processing. The above-mentioned observations were also confirmed by other researchers [20,44]. This is a positive effect, when taking into account the possibility of secondary pollution of water and soils with heavy metals and, as a consequence, their bioaccumulation in living organisms, especially in the case of agricultural use of sludge. Moreover, it must be stressed that processes used at WWTPs may significantly influence the sewage sludge characteristics, e.g., its pH value, the content of total and organic matter, etc. [8]. However, so far, there are not many publications on the heavy metal content and their chemical forms in sewage sludge, during the various stages of processing. Mostly these works are devoted to one selected type of sludge. For example, it was found that thickening of secondary sludge decreases the content of heavy metals in mobile fractions, compared to their content in the primary and secondary sludge [44]. The above findings are in good agreement with the results obtained in this study, as well as with the previous one [8]. Furthermore, the obtained results showed an increase in the content of heavy metals in mobile forms in the mixed sludge. This was probably caused by the decrease in pH and Eh values, compared to the sludge characteristics in the previous processing stages. Moreover, it was also found that anaerobic digestion and dehydration of sewage sludge reduced the mobility of heavy metals, which can minimize the level of potential risk posed by these elements. The probable reason was the increase of pH, during the processes that were conducted. Generally, according to the literature, the main factors affecting heavy metal availability from sludge are pH and Eh [45].

Table 8 presents the statistical summary data for pH, Eh, DM, and OM, as well as for heavy metals in the sewage sludge. The concentrations of metals below the limits of detection were set to zero. The obtained results indicate the presence of strong correlations between the content of Zn, Ni and Cu bound to the mobile/immobile fractions, and pH value; i.e., F2 (0.79) and F3 (0.88) for Zn and Ni, respectively, and F1 (−0.81), F4 (0.78) for Cu. Moreover, negative correlations were found between the concentration of Zn (F1) and Eh value (−0.92). The obtained results also indicate the presence of positive correlations between the content of Cr (F1) and DM (0.85), and Cr (F1) and OM (0.84). However, in this case, further analysis should be conducted (including seasonal changes in the physicochemical characteristic of sewage sludge). Moreover, there is some literature which confirms the existence of significant correlations between the selected physicochemical properties of sewage sludge and concentrations of easily mobile and available heavy metal fractions. For example, Wang et al. (2006) [44] found that Cu, Cr, and Pb in mobile fractions (F1+F2) were negatively correlated with the pH value of sludge collected at different stages of processing (primary sludge, active sludge, thickened sludge, digested sludge and dewatered sludge). In turn, other scientists indicated that in dewatered sludge from five different WWTPs, Ni and Pb (F2) were positively correlated with pH values. Moreover, they also found that Pb in mobile fractions (F1 and F2) was positively correlated with OM content, while and Zn (F1 and F2) negatively [21]. 

Based on the above results, it can be stated that this work paid special attention to the necessity of analyzing changes in the physicochemical properties of sewage sludge and concentrations of heavy metals, especially with regard to their chemical forms, in the whole technological line of the WWTP. This approach can have a significant impact on the proper assessment of the potential ecological risk associated with the presence of these elements in sewage sludge.

### 3.4. Assessment of Polluton Level and Ecological Risk

The assessment of the pollution level and potential ecological risk of selected heavy metals in sewage sludge from the “Central” WWTP in Bytom, was carried out using two groups of indices. The obtained results are shown in Table 9. 

The indices in the first group are focused on the total concentrations of heavy metals. The values of the Geoaccumulation Index (I_geo_) revealed that sewage sludge, at different stages of processing was moderately to heavily contaminated with Cd, Cu, and Hg (2.1–3.3; 2.0–2.9, and 1.5–3.5, respectively), moderately contaminated with Pb (1.4–2.0), and heavily contaminated with Zn (3.4–4.2). According to I_geo_, zinc posed the highest contamination in sewage sludge, while lead posed the lowest. In turn, the values of the Potential Ecological Risk Factor (ER), which additionally includes the toxicity of heavy metals, showed that level of ecological risk depended on sludge characteristics at different stages of processing. It indicated that mercury posed the highest potential risk (ER; 172.4–692.7) in sewage sludge (except S3). However, cadmium also posed a considerable to very high environmental risk (ER; 91.4–435.7). Moreover, the research also revealed that the activated sludge process (the most commonly applied biological process) carried out before introducing sludge to the secondary settling tank, as well as thickening (mechanical process assisted by the addition of a conditioning agent), had the greatest impact on the level of ecological risk associated with the presence of heavy metals in sewage sludge. Confirmation of the above fact is that heavy metals present in the secondary sludge posed the highest potential risk for the environment. The obtained results also revealed that mercury and cadmium are two of the major causes of sludge pollution, and at the same time may pose the highest potential ecological risk, despite their low content in the sewage sludge (at different stages of processing). In turn, zinc, which was the main pollutant in sewage sludge, did not pose a significant risk to the environment (except S3). Similar observations to those presented here were made by Li et al. (2015) [40] who found that dewatered sludge collected from three WWTPs (China) was moderately to heavily contaminated with Cd and Hg (I_geo_; 2.87–3.64, 2.14–2.75, for Cd and Hg, respectively), as well as moderately to very heavily contaminated by zinc (I_geo_; 2.41–3.07), where Cd and Hg posed a very high ecological risk (ER; 495.0–842.14 and 369.36–603.63 for Cd and Hg, respectively), while zinc posed only moderate to considerable risk (ER; 11.94–18.92). The above results were partly confirmed by Yang et al. (2017) [17] who indicated that sewage sludge collected from four selected WWTPs (China) were extremely contaminated with Cd (I_geo_; 6.53–7.18), which posed a very high environmental risk (ER; 4150.86–6521.55), despite its low content in sewage sludge. Also, Duan et al. (2017b) [11] confirmed that according to I_geo_ and ER values, sludge was moderately to heavily contaminated with Cd (I_geo_; 1.76–3.46) and posed a potential environmental threat (ER; 79.42–256.62). Whereas, in contrast to the above-mentioned results, other researchers who analyzed sewage sludge from five selected WWTPs (Poland) indicated that cadmium posed a low ecological risk [21]. This confirms that every type of sludge is different, and its characteristics depend on the processes used in WWTPs, as well as the nature of the treated wastewater [46]. Therefore, each type of sludge should be considered individually.

The second group of analyzed indices refers only to the chemical forms of heavy metals in sewage sludge. In accordance with the values of the Individual Contamination Factor (ICF), it was shown that regardless of the sampling point, sewage sludge was moderately to very highly polluted with Zn (ICF; 14.4–63.0), Cu (ICF; 9.2–24.7), Cd (ICF; 1.6–3.1), Cr (ICF; 1.2–2.1), and Ni (ICF; 1.1–1.3). It was also indicated that in accordance with the modified Risk Assessment Code (RAC_m_), the highest potential ecological risk may be posed by Zn (RAC_m_; 55.0–70.4%), Ni (RAC_m_; 27.0–40.8%), and Cd (RAC_m_; 23.4–51.0%). The above observations were partly confirmed by the Ecological Risk Factor (ERF)—author’s index, whose values indicated that Zn, Ni, and Cd posed a potential environmental threat. Similar results were obtained by researchers who analyzed sewage sludge from several WWTPs, and found that Zn posed a high ecological risk, while Cu and Cd posed only medium threats [17]. In turn, other scientists found that heavy metals in selected sewage sludge posed low to very high ecological risks, i.e., Zn (high to very high), Cu (low), Ni (high), Cd (medium), and Cr (low) [40].

In summary, according to the I_geo_ and ER values, heavy metals in dewatered sludge (S7) were ranked in the following order: Zn > Hg > Cd > Cu > Pb > Ni > Cr and Hg > Cd > Cu > Pb > Zn > Ni > Cr, respectively. In turn, according to ICF, RAC_m_, and ERF: Zn > Cu > Cd > Cr, Ni > Pb; Zn > Ni > Cd > Cr > Cu > Pb and Zn > Ni > Cd > Cr, Cu, Pb, respectively. Moreover, the research conducted also revealed that processes used in the WWTP analyzed enabled the agricultural or natural use of sewage sludge, but they did not eliminate the ecological risk associated with the presence of heavy metals. Therefore, taking into account that these toxic elements may still accumulate in plants and cause health problems to humans and animals, or undergo leaching from soils to ground water, it is necessary to introduce methods which allow their content in wastewater to be reduced, and as a consequence also their content in sewage sludge. Among these methods, we can distinguish the natural process of biofiltration performed by invasive bivalves. This process is environmentally friendly and consumes less energy than precipitation, ion exchange, or membrane separation. Moreover, bivalves are aquatic organisms, which are characterized by relative tolerance to toxic substances, and have high biofiltration, as well as bioaccumulation features. They can be used for water disinfection, wastewater treatment, etc. After the process has been completed, the contaminated mollusks may be incinerated or stored in special landfills [47]. There are some studies focused on the bioaccumulation of heavy metals by bivalves from different environmental samples. For example, Rosa et al. (2014) [48] used *C. fluminea* for the removal of metals (Al, Cd, Co, Cu, Fe, Mn, Ni, Pb, and Zn) from acid mine drainage. The experiment conducted showed that most of the metals removed from the water column were detected in the bivalves' shells. Other researchers examined the ability of *D. polymorpha* for removing selected metals (Al, Cr, Cu, Fe, Mn, Ni, and Pb) from municipal effluent. The obtained results revealed that the highest percentage removed was obtained for Cr, while the lowest was obtained for Ni (Magni et al., 2015) [49]. It must be emphasized that the above described method was applied for the removal of toxic elements from treated wastewater. However, the results presented here encourage conducting further research related to the possibility of using bivalves as a natural biofilter for removing metals from untreated (raw) wastewater. This approach can contribute to reducing the total content of heavy metals in the sewage sludge. Moreover, this also may constitute the next step toward minimizing the level of ecological risk associated with the presence of heavy metals in sewage sludge.

## 4. Conclusions

The analysis of total heavy metal concentrations and their chemical forms in sewage sludge is one of the most important issues in terms of assessment of the potential risk posed by these elements to the environment, and hence to living organisms. This is particularly important in regions of high heavy metal pollution. It has been found that concentrations of heavy metals examined in sewage sludge do not exceed the permissible norms in Poland and the EU, which is favorable in terms of agricultural use of sludge, and has a significant impact, both on the environment quality and human health. The analysis of sludge characteristics at different stages of processing revealed that biological processes and thickening of sewage sludge have a significant impact on heavy metal distribution, while anaerobic digestion and dehydration decrease the mobility of metals. The risk analysis conducted according to total and speciation indices showed that Zn, Cd, Ni, and Hg posed the highest ecological risk, among the analyzed heavy metals. The comparison of values of different indices showed that when taking into account the chemical forms of heavy metals, in some cases they can be hazardous to the environment, and in some cases not. This can be seen when comparing the values of the Geoaccumulation Index (I_geo_) and the Individual Contamination Factor (ICF) in relation to Cr, Ni, and Pb. A similar relationship was observed for the values of the Potential Ecological Risk Factor (ER), modified Risk Assessment Code (RAC_m_), and Ecological Risk Factor (ERF) in relation to Zn and Ni. The above findings confirmed that knowledge of the total concentrations of heavy metals allows only the sludge pollution level to be assessed whereas the mobility, bioavailability, and toxicity of these elements depend on the chemical forms in which they occur. Therefore, both total concentration and the chemical form of heavy metals in sewage sludge should be monitored and controlled regularly in order to identify potential hazards posed by these elements. This approach will allow for leading new regulations in this respect, which will contribute to the reduction of risk associated with secondary pollution of soils with heavy metals, as a result of their fertilization with sewage sludge. Moreover, it is also necessary to pay special attention to the need for the implementation of methods which allow for the removal of heavy metals at the very beginning of a WWTP’s technological line, i.e., from raw wastewater. This could contribute to lowering the content of metals in sewage sludge. 

## Figures and Tables

**Table 1:** The operational parameters of the WWTP.

Parameter	Unit	Value
Population equivalent (PE)	-	143,368
Average flow (Q)	m^3^·d^−1^	19,330
Hydraulic retention time (HRT) of sludge in anaerobic digester	d	21
Temperature in the anaerobic digester	°C	36

Data for the year 2017 obtained from the “Central” WWTP in Bytom.

**Table 2:** Scheme of the Community Bureau of Reference (BCR) sequential extraction procedure [8,19,20].

	Fraction	BCR Procedure
F1	Acid soluble/exchangeable fraction; bound to carbonates (mobile)	Add 20 mL of 0.11 mol·L^−1^ acetic acid (CH_3_COOH) to 0.5 g of sludge sample. Shake for 16-h.
F2	Reducible fraction; bound to Mn and Fe oxides (mobile)	Add 20 mL 0.1 mol·L^−1^ hydroxylamine hydrochloride (NH_2_OH·HCl; adjusted with HNO_3_ to pH = 2) to residue from first step of extraction. Shake for 16 h.
F3	Oxidizable fraction; bound to organic matter and sulfides (immobile)	Add 5 mL of 8.8 mol·L^−1^ hydrogen peroxide (H_2_O_2_) and incubate at 85 °C for 1 h (repeat the procedure twice). Afterwards, add 25 mL of 1 mol L^−1^ ammonium acetate (CH_3_COONH_4_; adjusted with HNO_3_ to pH = 2) to residue from second step of extraction. Shake for 16 h.
F4	Residual fraction (immobile)	Add 5 ml HNO_3_ and 15 mL HCl to residue from third step of extraction.

**Table 3:** The pollution level and ecological risk criteria for heavy metals.

Indices	Equation with Description	Category	Description and Abbreviations
Geoaccumulation Index (I_geo_) [24]	$I_{geo}=log_{2}\left(\frac{C_{n}}{1.5B_{n}}\right)$ C_n_—measured concentration of metal in the sludge sample; B_n_—geochemical background value in the Earth’s crust [38]	I_geo_ ≤ 0 0 < I_geo_ ≤ 1 1 < I_geo_ ≤ 2 2 < I_geo_ ≤ 3 3 < I_geo_ ≤ 4 4 < I_geo_ ≤ 5 5 < I_geo_	Practically uncontaminated (PUC) Uncontam. to moderately contam. (U-MC) Moderately contaminated (MC) Moderately to heavily contam. (M-HC) Heavily contaminated (HC) Heavily to extremely contam. (H-EC) Extremely contaminated (EC)
Potential Ecological Risk Factor (ER) [25]	$ER=T_{f}^{i}C_{f}$ $T_{f}^{i}$ —the toxic response factor of metal; C_f_—single metal pollution factor	ER ≤ 40 40 < ER ≤ 80 80 < ER ≤ 160 160 < ER ≤ 320 ER > 320	Low risk (LR) Moderate risk (MR) Considerable risk (CR) High risk (HR) Very high risk (VHR)
Individual Contamination Factor (ICF) [23,26]	$ICF\;=\frac{F_{1}+F_{2}+F_{3}}{F_{4}}$ F_1_, F_2_, F_3,_ F_4_—the content of metal in exchangeable, reducible, oxidizable, and residual fraction	ICF ≤ 1 1 < ICF ≤ 3 3 < ICF ≤ 6 ICF > 6	Low contamination (LC) Moderate contamination (MC) Considerable contamination (CC) Very high contamination (VHC)
modified Risk Assessment Code (RAC_m_) (based on criteria specified in reference [27])	${RAC}_{m}=F_{1}+F_{2}$ F_1_, F_2_—the percentage share of metal in exchangeable and reducible fractions	RAC_m_ ≤ 1% 1% < RAC_m_ ≤ 10% 10% < RAC_m_ ≤ 30% 30% < RAC_m_ ≤ 50% 50% < RAC_m_	No risk (NR) Low risk (LR) Medium risk (MR) High risk (HR) Very high risk (VHR)
Ecological Risk Factor (ERF)—author’s index (this study)	$ERF\;=\frac{F_{1}+F_{2}}{F_{3}+F_{4}}$ F_1_, F_2_, F_3_, F_4_—the content of metal in exchangeable, reducible, oxidizable, and residual fractions	0 < ERF ≤ 0.4 0.4 < ERF ≤ 1 1 < ERF	Low risk (LR) Medium risk (MR) High risk (HR)

**Table 4:** Physicochemical characteristics of sewage sludge.

Sampling Points	pH	Eh	DM	OM	Moisture
		mV	%	%_DM_	%
S1	7.4	−175	0.6	83.3	99.4
S2	5.4	−350	6.5	78.5	97.5
S3	7.4	−188	0.9	77.8	99.1
S4	7.1	−177	7.1	70.4	92.8
S5	5.7	−273	5.4	75.9	94.6
S6	6.7	−343	3.4	61.8	96.6
S7	7.4	−272	20.3	64.0	79.7

**Table 5:** Total concentrations of heavy metals in sewage sludge.

Metal	S1	S2	S3	S4	S5	S6	S7
	mg·kg^−1^						
**Cd**	2.7 ± 0.1	1.8 ± 0.2	3.0 ± 0.2	3.1 ± 0.4	2.3 ± 0.1	4.0 ± 0.2	4.1 ± 0.8
**Cr**	57.3 ± 8.7	34.9 ± 3.7	54.8 ± 6.0	62.7 ± 5.9	53.4 ± 16.5	68.3 ± 2.4	67.1 ± 8.8
**Cu**	123.6 ± 13.1	104.1 ± 20.2	138.3 ± 11.4	143.0 ± 26.2	117.7 ± 2.4	188.9 ± 12.5	194.0 ± 45.0
**Ni**	55.0 ± 21.1	51.2 ± 18.8	62.0 ± 14.8	64.1 ± 22.0	58.7 ± 22.7	98.1 ± 21.0	95.2 ± 20.2
**Pb**	123.5 ± 1.3	97.6 ± 5.6	137.7 ± 12.8	141.0 ± 13.6	123.7 ± 6.7	189.2 ± 6.2	187.8 ± 20.4
**Zn**	1429.5 ± 41.8	1092.2 ± 63.3	1641.0 ± 41.4	1558.1 ± 87.0	1407.8 ± 80.4	1847.1 ± 30.2	1851.6 ± 53.2
**Hg**	1.0 ± 2.8	0.3 ± 1.3	0.7 ± 1.6	0.5 ± 4.0	1.0 ± 3.5	1.1 ± 3.7	1.0 ± 1.3
**Sum**	1792.6	1382.1	2037.5	1972.5	1764.6	2396.7	2400.8

Results are expressed as the mean ± standard deviation in mg kg^−1^ of dry matter.

**Table 6:** Pearson’s correlation coefficients between heavy metals in sewage sludge.

	Cd	Cr	Cu	Ni	Pb	Zn	Hg
Cd	1.0000						
Cr	**0.9098**	1.0000					
Cu	**0.9823**	**0.8430**	1.0000				
Ni	**0.9287**	**0.7703**	**0.9780**	1.0000			
Pb	**0.9825**	**0.8811**	**0.9925**	**0.9752**	1.0000		
Zn	**0.9716**	**0.9245**	**0.9359**	**0.8767**	**0.9579**	1.0000	
Hg	0.5109	0.6440	0.4734	0.4937	0.5534	0.5940	1.0000

Bold—significant correlations at *p* < 0.05.

**Table 7:** Heavy metal concentrations in individual fractions of sewage sludge.

Metal	Fraction	S1	S2	S3	S4	S5	S6	S7
mg·kg^−1^_DM_								
**Cd**	F1	BDL	BDL	BDL	BDL	BDL	BDL	BDL
	F2	0.9 ± 0.0	1.1 ± 0.0	1.2 ± 0.1	0.9 ± 0.1	1.1 ± 0.0	1.3 ± 0.0	1.1 ± 0.1
	F3	1.2 ± 0.3	1.1 ± 0.2	1.2 ± 0.1	1.3 ± 0.1	1.1 ± 0.3	1.9 ± 0.1	2.2 ± 0.2
	F4	0.8 ± 0.1	BDL	1.1 ± 0.4	1.4 ± 0.2	0.7 ± 0.0	1.8 ± 0.0	1.5 ± 0.1
	R, %	106.8	124.4	112.2	114.1	126.7	123.6	119.2
**Cr**	F1	BDL	BDL	BDL	BDL	0.6 ± 0.0	BDL	1.2 ± 0.0
	F2	BDL	BDL	BDL	BDL	BDL	BDL	BDL
	F3	19.7 ± 8.2	17.0 ± 4.5	23.5 ± 1.4	22.1 ± 0.4	17.7 ± 1.6	25.2 ± 0.5	25.5 ± 0.7
	F4	16.0 ± 0.6	8.0 ± 0.8	15.3 ± 1.3	19.0 ± 2.6	12.6 ± 0.5	24.8 ± 0.7	22.2 ± 0.2
	R, %	62.3	71.6	70.9	65.5	58.0	73.3	73.0
**Cu**	F1	0.7 ± 0.1	2.3 ± 0.1	0.8 ± 0.1	0.9 ± 0.0	1.9 ± 0.0	0.5 ± 0.1	1.4 ± 0.1
	F2	4.8 ± 1.2	6.9 ± 2.4	1.6 ± 0.2	1.1 ± 0.1	3.5 ± 05	0.7 ± 0.1	1.7 ± 0.1
	F3	118.3 ± 30.4	110.8 ± 23.1	141.1 ± 7.0	151.7 ± 5.6	126.9 ± 14.8	210.5 ± 3.8	198.8 ± 6.3
	F4	13.4 ± 2.1	4.9 ± 0.5	11.1 ± 1.6	12.1 ± 0.4	7.6 ± 0.5	16.5 ± 0.4	14.5 ± 0.5
	R; %	110.9	120.0	111.8	116.0	118.8	120.8	111.5
**Ni**	F1	5.4 ± 0.2	5.4 ± 0.2	5.8 ± 0.9	5.6 ± 0.2	7.6 ± 0.1	9.4 ± 0.0	11.2 ± 0.1
	F2	3.6 ± 0.1	1.8 ± 0.0	3.7 ± 0.2	3.6 ± 0.2	2.6 ± 0.1	4.7 ± 0.1	2.9 ± 0.0
	F3	8.3 ± 8.9	4.2 ± 2.8	8.2 ± 1.1	9.5 ± 0.1	3.7 ± 0.1	7.3 ± 0.3	7.1 ± 0.4
	F4	13.9 ± 1.1	10.9 ± 4.0	13.2 ± 2.3	15.2 ± 3.3	11.1 ± 0.4	19.1 ± 2.4	18.0 ± 0.8
	R; %	56.9	43.7	49.9	52.9	42.5	41.4	41.1
**Pb**	F1	BDL	BDL	BDL	1.1 ± 0.7	BDL	BDL	BDL
	F2	BDL	1.2 ± 0.1	BDL	BDL	BDL	BDL	BDL
	F3	18.0 ± 14.8	19.5 ± 10.0	7.9 ± 1.0	3.8 ± 0.3	10.2 ± 0.6	13.3 ± 0.4	19.4 ± 1.0
	F4	65.3 ± 4.8	24.3 ± 2.0	74.5 ± 10.7	80.3 ± 14.2	52.8 ± 4.9	107.9 ± 1.1	93.9 ± 5.5
	R; %	67.4	46.1	59.5	60.4	50.9	64.1	60.3
**Zn**	F1	123.0 ± 6.6	308.1 ± 10.2	137.4 ± 17.0	94.1 ± 4.8	220.9 ± 3.5	230.5 ± 7.4	252.5 ± 4.9
	F2	538.1 ± 11.3	334.2 ± 10.8	555.6 ± 23.1	568.9 ± 20.6	482.1 ± 15.4	600.5 ± 6.7	545.1 ± 10.4
	F3	304.7 ± 187.2	256.2 ± 135.0	377.3 ± 28.8	455.6 ± 34.6	285.5 ± 30.9	560.1 ± 20.3	571.3 ± 44.2
	F4	57.5 ± 6.7	14.3 ± 2.5	59.1 ± 10.0	70.0 ± 12.2	34.6 ± 3.0	96.5 ± 1.9	81.8 ± 3.2
	R; %	71.6	83.6	68.8	76.3	72.7	80.5	78.3

Results are expressed as the mean ± standard deviation in mg kg^−1^ of dry matter; R—heavy metal recovery rate calculated by comparing the sum of the four fractions with the total concentrations of heavy metals in the sludge samples; BDL—below detection limit.

**Table 8:** Pearson’s correlation coefficients between parameters of sewage sludge and concentrations of heavy metal fractions.

Cd	F1	F2	F3	F4
ph	-*	−0.2487	0.3950	0.6567
Eh	-*	−0.6903	−0.3144	0.1519
DM	-*	0.0675	0.6888	0.2389
OM	-*	0.0493	0.6342	0.1709
Cr	F1	F2	F3	F4
pH	0.0624	-*	0.7280	0.6414
Eh	−0.1557	-*	0.0872	0.0460
DM	**0.8499**	-*	0.3444	0.3007
OM	**0.8356**	-*	0.2720	0.2303
Cu	F1	F2	F3	F4
ph	**−0.8119**	−0.5937	0.4028	**0.7781**
Eh	−0.4887	−0.2252	−0.2829	0.2106
DM	0.3224	−0.1630	0.4778	0.1323
OM	0.3957	−0.1056	0.4206	0.0513
Ni	F1	F2	F3	F4
ph	0.1668	0.6162	**0.8836**	0.5867
Eh	−0.4328	0.2535	0.6893	−0.1007
DM	0.7112	−0.3795	−0.1092	0.3833
OM	0.6677	−0.4475	−0.1637	0.3164
Pb	F1	F2	F3	F4
ph	0.1932	−0.6909	−0.1555	0.6942
Eh	0.4488	−0.5595	−0.4874	0.1307
DM	0.0486	0.0129	0.3426	0.2183
OM	0.0694	0.0776	0.3361	0.1439
Zn	F1	F2	F3	F4
ph	−0.6627	**0.7872**	0.5452	0.7196
Eh	**−0.9238**	0.4600	−0.1032	0.1377
DM	0.4299	−0.0416	0.5198	0.2137
OM	0.4601	−0.1153	0.4616	0.1389

Bold—significant correlations at *p* < 0.05; * Concentration of heavy metal below the limit of detection at all sampling points (no statistical analysis).

**Table 9:** Results of heavy metal pollution level and potential ecological risk in sewage sludge (bold indicates the highest levels).

	Index	Cd	Cr	Cu	Ni	Pb	Zn	Hg
S1	I_geo_	**2.7 (M-HC)**	−1.3	**2.3 (M-HC)**	0.0	1.4 (MC)	**3.8 (HC)**	**3.5 (HC)**
	ER	**293.6 (HR)**	1.2	35.7	7.4	19.3	21.0	**664.8 (VHR)**
	ICF	**2.6 (MC)**	**1.2 (MC)**	**9.2 (VHC)**	**1.3 (MC)**	0.3	**16.8 (VHC)**	-
	RAC_m_	**32.0 (HR)**	0.0	4.0	**29.0 (MR)**	0.0	**64.6 (VHR)**	-
	ERF	**0.5 (MR)**	0.0	0.0	0.4	0.0	**1.8 (HR)**	-
S2	I_geo_	**2.1 (M-HC)**	−2.0	**2.0 (MC)**	−0.1	1.0	**3.4 (HC)**	**1.5 (MC)**
	ER	**189.0 (HR)**	0.7	30.1	6.9	15.2	16.0	**172.4 (HR)**
	ICF	0.0	**2.1 (MC)**	**24.7 (VHC)**	**1.1 (MC)**	0.9	**63.0 (VHC)**	-
	RAC_m_	**51.0 (VHR)**	1.7	7.4	**32.4 (HR)**	2.7	**70.4 (VHR)**	-
	ERF	**1.0 (MR)**	0.0	0.1	**0.5 (MR)**	0.0	**2.4 (HR)**	-
S3	I_geo_	**2.9 (M-HC)**	−1.4	**2.4 (M-HC)**	0.2	**1.5 (MC)**	**4.0 (HC)**	**3.0 (M-HC)**
	ER	**91.4 (CR)**	**109.5 (CR)**	**691.4 (VHR)**	**310.2 (HR)**	**688.5 (VHR)**	**1641.0 (VHR)**	29.1
	ICF	**2.1 (MC)**	**1.5 (MC)**	**12.9 (VHC)**	**1.3 (MC)**	0.1	**18.1 (VHC)**	-
	RAC_m_	**35.5 (HR)**	0.0	1.5	**30.9 (HR)**	0.0	**61.4 (VHR)**	-
	ERF	**0.5 (MR)**	0.0	0.0	0.4	0.0	**1.6 (HR)**	-
S4	I_geo_	**2.9 (M-HC)**	−1.2	**2.5 (M-HC)**	0.2	**1.6 (MC)**	**3.9 (HC)**	**2.3 (M-HC)**
	ER	**337.4 (VHR)**	1.3	**41.3 (MR)**	8.7	22.0	22.9	**301.0 (HR)**
	ICF	**1.6 (MC)**	**1.2 (MC)**	**12.7 (VHC)**	**1.2 (MC)**	0.1	**16.0 (VHC)**	-
	RAC_m_	**25.0 (MR)**	0.0	1.2	**27.0 (MR)**	1.2	**55.8 (VHR)**	-
	ERF	0.3	0.0	0.0	0.4	0.0	**1.3 (HR)**	-
S5	I_geo_	**2.5 (M-HC)**	−1.4	**2.2 (M-HC)**	0.1	**1.4 (MC)**	**3.8 (HC)**	**3.5 (HC)**
	ER	**251.4 (HR)**	1.1	34.0	7.9	19.3	20.7	**684.4 (VHR)**
	ICF	**3.1 (CC)**	**1.5 (MC)**	**17.4 (VHC)**	**1.3 (MC)**	0.2	**28.6 (VHC)**	-
	RAC_m_	**37.7 (HR)**	2.0	3.8	**40.8 (HR)**	0.0	**68.7 (VHR)**	-
	ERF	**0.6 (MR)**	0.0	0.0	**0.7 (MR)**	0.0	**2.2 (HR)**	-
S6	I_geo_	**3.3 (HC)**	−1.1	**2.9 (M-HC)**	0.8	**2.0 (MC)**	**4.2 (HC)**	**3.5 (HC)**
	ER	**431.5 (VHR)**	1.4	**54.6 (MR)**	13.3	29.6	27.1	**692.7 (VHR)**
	ICF	**1.8 (MC)**	1.0	**12.8 (VHC)**	**1.1 (MC)**	0.1	**14.4 (VHC)**	-
	RAC_m_	**25.3 (MR)**	0.0	0.5	**34.8 (HR)**	0.0	**55.9 (VHR)**	-
	ERF	0.3	0.0	0.0	**0.5 (MR)**	0.0	**1.3 (HR)**	-
S7	I_geo_	**3.3 (HC)**	−1.1	**2.9 (M-HC)**	0.8	**2.0 (MC)**	**4.2 (HC)**	**3.4 (HC)**
	ER	**435.7 (VHR)**	1.4	**56.1 (MR)**	12.9	29.4	27.2	**627.2 (VHR)**
	ICF	**2.2 (MC)**	**1.2 (MC)**	**13.9 (VHC)**	**1.2 (MC)**	0.2	**16.7 (VHC)**	-
	RAC_m_	**23.4 (MR)**	2.4	1.4	**35.9 (HR)**	0.0	**55.0 (VHR)**	-
	ERF	0.3	0.0	0.0	**0.6 (MR)**	0.0	**1.2 (HR)**	-
