# Correction: Tytła, M. Assessment of Heavy Metal Pollution and Potential Ecological Risk in Sewage Sludge from Municipal Wastewater Treatment Plant Located in the Most Industrialized Region in Poland—Case Study. *Int. J. Environ. Res. Public Health* 2019, *16*, 2430

**DOI:** 10.3390/ijerph20105795

**Published:** 2023-05-11

**Authors:** Malwina Tytła

**Affiliations:** Institute of Environmental Engineering, Polish Academy of Sciences, 34 M. Skłodowskiej-Curie St., 41-819 Zabrze, Poland; malwina.tytla@ipis.zabrze.pl

**Missing Funding**

In the original publication, the number of grant “DMN/2018” was not included. Thus, the correct Funding statement is the following:

**Funding**: This research was founded by the Ministry of Science and Higher Education, Poland, under project number DMN8/2018. The research was carried out at the Institute of Environmental Engineering, Polish Academy of Sciences in Zabrze.

**Text Correction**

There was an error in the original publication [1]. The information about the modification of Risk Assessment Code index was not included (RAC_m_). A correction has been made to name and symbol of the above-mentioned index. The name of the risk index should be “modified Risk Assessment Code (RAC_m_)” instead of “Risk Assessment Code (RAC)”. The missing information and/or explanation are included through all parts of the publication. The changes are as follows:

(1) **A correction has been made to “Abstract”, first paragraph, fourth sentence:**

The corrected sentence appears below.

To assess the pollution level and potential ecological risk, the following indices were used: Geoaccumulation Index (I_geo_), Potential Ecological Risk Factor (ER), Individual Contamination Factor (ICF), modified Risk Assessment Code (RAC_m_), and Ecological Risk Factor (ERF)—the author’s index.

(2) **A correction has been made to Section 1. (“Introduction”), fourth paragraph, third and fifth sentence**.

The corrected sentences appear below.

**Section 1, fourth paragraph, third sentence:**

The first group, total content, comprised the Geoaccumulation Index (I_geo_) [24] and Potential Ecological Risk Factor (ER) [25], whereas the second group, speciation indices, comprised the Individual Contamination Factor (ICF) [23,26], modified Risk Assessment Code (RAC_m_) (based on criteria specified in reference) [27], and Ecological Risk Factor (ERF)—the author’s index. 

**Section 1, fourth paragraph, fifth sentence:**

For example, I_geo_ considers the accumulation level of heavy metals in sewage sludge without toxicity impact; ER focuses both on the total quantity of heavy metals and toxicity, while ICF, RAC_m_, and ERF focus on heavy metal mobility [11].

(3) **A correction has been made to Section 2.3. (“Pollution Level and Ecological risk”), first paragraph.** The missing information about the modification of RAC index was included.

The corrected paragraph appears below.

To assess the pollution level and potential ecological risk of heavy metals in sewage sludge, the following indices were used: Geoaccumulation Index (I_geo_) [24], Potential Ecological Risk Factor (ER) [25], Individual Contamination Factor (ICF) [23,26], modified Risk Assessment Code (RAC_m_) (based on criteria specified in reference [27]) and Ecological Risk Factor (ERF)—the author’s index. The I_geo_ and ER indices refer to total concentrations of heavy metals, while ICF, RAC_m_, and ERF refer to their chemical forms. The pollution levels and ecological risks of heavy metals in sewage sludge were calculated by the equations shown in Table 3. However, the observations made so far have shown that some metals are characterized by a low or moderately low percentage share in the most mobile fraction F1, such as Cu or Zn, or they do not show this share at all, such as Cd, and yet still by their share in fraction F2 indicate the existence of a potential threat to the environment (confirmed by the other ecological risk indices). In view of the above, it was decided to modify the original formula for calculating the RAC index. The original formula took into account the percentage share of a single metal only in the first fraction (F1). In turn, the proposed modification consisted in taking into account the share of a given metal also in the second fraction (F2). The category and description have remained unchanged.

(4) **A correction has been made to Section 3.4. (“Assessment of Pollution Level and Ecological Risk”), third (third sentence) and fourth paragraph (second sentence).**

The corrected sentences appear below.

**Section 3.4, third paragraph, third sentence:**

It was also indicated that in accordance with the modified Risk Assessment Code (RAC_m_), the highest potential ecological risk may be posed by Zn (RAC_m_; 55.0–70.4%), Ni (RAC_m_; 27.0–40.8%), and Cd (RAC_m_; 23.4–51.0%). 

**Section 3.4, fourth paragraph, second sentence:**

In turn, according to ICF, RAC_m_, and ERF: Zn > Cu > Cd > Cr, Ni > Pb; Zn > Ni > Cd > Cr > Cu > Pb and Zn > Ni > Cd > Cr, Cu, Pb, respectively.

(5) **A correction has been made to Section 4. (”Conclusions”), first paragraph, eighth sentence.**

The corrected sentence appears below.

A similar relationship was observed for the values of the Potential Ecological Risk Factor (ER), modified Risk Assessment Code (RAC_m_), and Ecological Risk Factor (ERF) in relation to Zn and Ni.

**Other Text Correction**

There was an error in the original publication [1]. 

**A correction has been made to Section 3.4. (“Assessment of Pollution Level and Ecological Risk”), third paragraph, sixth sentence.** There should be“Cu (low)” instead of “Cu (very high)”. 

The corrected sentence appears below.

In turn, other scientists found that heavy metals in selected sewage sludge posed low to very high ecological risks, i.e., Zn (high to very high), Cu (low), Ni (high), Cd (medium), and Cr (low) [40].

**A correction has been made to Section 1. (“Introduction”), third paragraph, sixth sentence, as well as to Section 3.2. (“Total Heavy Metal Concentrations”), first paragraph, eighth sentence.** There should be “Regulation of the Minister of Environment of 6 February 2015 on municipal sewage sludge J. L. 2015, item. 257” instead of “Regulation of the Minister of Environment of 13 July 2010 on municipal sewage sludge J. L. 2010, No. 137, item. 924”. 

The corrected sentences appear below:

**Section 1, third paragraph, sixth sentence:**

In Poland, one of the most important criteria for agricultural use of sludge is heavy metal concentrations; for which limits are regulated by the Regulation of the Minister of Environment of 6th February 2015 on municipal sewage sludge (J. L. 2015, item. 257) [15], being compatible with the Council Directive of 12 June 1986 on the protection of the environment, and in particular of the soil, when sewage sludge is used in agriculture (86/278/EEC) [16].

**Section 3.2, first paragraph, eighth sentence.**

However, in this study, the total concentrations of heavy metals did not exceed the permissible standards for sewage sludge in Poland (J. L. 2015, item. 257) [15] and EU (86/278/EEC) [16].

**Error in Table**

**A correction has been made in Table 3.**

In the original publication [1], there was a mistake in Table 3. The name of the the risk index should be “modified Risk Assessment Code (RAC_m_)” instead of “Risk Assessment Code (RAC)” (column numbers 1–3, row number 5). The name of chemical fraction should be “exchangeable” instead of “exchangeable/carbonate” (column number 2, row numbers 4–6). The order of chemical fractions should be “exchangaeable, reducible, oxidizable and residual” instead of “exchangaeable, oxidizable, reducible and residual” (column number 2, row numbers 4–6). The incorrect abbreviation of risk index, it should be “ICF” instead of “CF” (column number 3, row number 4). The words “uncontaminated” and “contaminated” should be replaced with “uncontam.” and “contam.”, respectively (column number 4, row number 2).

The corrected Table 3 appears below.

**Table 3:** The pollution level and ecological risk criteria for heavy metals.

Indices	Equation with Description	Category	Description and Abbreviations
Geoaccumulation Index (I_geo_) [24]	$I_{geo}={log}_{2}\left(\frac{C_{n}}{1.5B_{n}}\right)$ C_n_—measured concentration of metal in the sludge sample; B_n_—geochemical background value in the Earth’s crust [38]	I_geo_ ≤ 0 0 < I_geo_ ≤ 1 1 < I_geo_ ≤ 2 2 < I_geo_ ≤ 3 3 < I_geo_ ≤ 4 4 < I_geo_ ≤ 5 5 < I_geo_	Practically uncontaminated (PUC) Uncontam. to moderately contam. (U-MC) Moderately contaminated (MC) Moderately to heavily contam. (M-HC) Heavily contaminated (HC) Heavily to extremely contam. (H-EC) Extremely contaminated (EC)
Potential Ecological Risk Factor (ER) [25]	${ER=T}_{f}^{i}C_{f}$ $T_{f}^{i}$—the toxic response factor of metal; C_f_—single metal pollution factor	ER ≤ 40 40 < ER ≤ 80 80 < ER ≤ 160 160 < ER ≤ 320 ER > 320	Low risk (LR) Moderate risk (MR) Considerable risk (CR) High risk (HR) Very high risk (VHR)
Individual Contamination Factor (ICF) [23,26]	$ICF=\frac{F_{1}+F_{2}+F_{3}}{F_{4}}$ F_1_, F_2_, F_3,_ F_4_—the content of metal in exchangeable, reducible, oxidizable, and residual fraction	ICF ≤ 1 1 < ICF ≤ 3 3 < ICF ≤ 6 ICF > 6	Low contamination (LC) Moderate contamination (MC) Considerable contamination (CC) Very high contamination (VHC)
modified Risk Assessment Code (RAC_m_) (based on criteria specified in reference [27])	*RAC_m_* = *F_1_ + F_2_* F_1_, F_2_—the percentage share of metal in exchangeable and reducible fractions	RAC_m_ ≤ 1% 1% < RAC_m_ ≤ 10% 10% < RAC_m_ ≤ 30% 30%< RAC_m_ ≤ 50% 50% < RAC_m_	No risk (NR) Low risk (LR) Medium risk (MR) High risk (HR) Very high risk (VHR)
Ecological Risk Factor (ERF)—author’s index (this study)	$ERF=\frac{F_{1}+F_{2}}{F_{3}+F_{4}}$ F_1_, F_2_, F_3_, F_4_—the content of metal in exchangeable, reducible_,_ oxidizable, and residual fractions	0 < ERF ≤ 0.4 0.4 < ERF ≤ 1 1 < ERF	Low risk (LR) Medium risk (MR) High risk (HR)

**A correction has been made in Table 9.**

In the original publication [1], there was a mistake in Table 9. The name of the the risk index should be “modified Risk Assessment Code (RAC_m_)” instead of “Risk Assessment Code (RAC)” (column number 1, row numbers 2–8). The lack of explanation and no bold font, i.e., “109.5 (CR)” of the level of ER index in relation to Cr at the sampling point S3. 

The corrected Table 9 appears below.

**Table 9:** Results of heavy metal pollution level and potential ecological risk in sewage sludge (bold indi-cates the highest levels).

	Index	Cd	Cr	Cu	Ni	Pb	Zn	Hg
S1	I_geo_	**2.7 (M-HC)**	−1.3	**2.3 (M-HC)**	0.0	1.4 (MC)	**3.8 (HC)**	**3.5 (HC)**
	ER	**293.6 (HR)**	1.2	35.7	7.4	19.3	21.0	**664.8 (VHR)**
	ICF	**2.6 (MC)**	**1.2 (MC)**	**9.2 (VHC)**	**1.3 (MC)**	0.3	**16.8 (VHC)**	-
	RAC_m_	**32.0 (HR)**	0.0	4.0	**29.0 (MR)**	0.0	**64.6 (VHR)**	-
	ERF	**0.5 (MR)**	0.0	0.0	0.4	0.0	**1.8 (HR)**	-
S2	I_geo_	**2.1 (M-HC)**	−2.0	**2.0 (MC)**	−0.1	1.0	**3.4 (HC)**	**1.5 (MC)**
	ER	**189.0 (HR)**	0.7	30.1	6.9	15.2	16.0	**172.4 (HR)**
	ICF	0.0	**2.1 (MC)**	**24.7 (VHC)**	**1.1 (MC)**	0.9	**63.0 (VHC)**	-
	RAC_m_	**51.0 (VHR)**	1.7	7.4	**32.4 (HR)**	2.7	**70.4 (VHR)**	-
	ERF	**1.0 (MR)**	0.0	0.1	**0.5 (MR)**	0.0	**2.4 (HR)**	-
S3	I_geo_	**2.9 (M-HC)**	−1.4	**2.4 (M-HC)**	0.2	**1.5 (MC)**	**4.0 (HC)**	**3.0 (M-HC)**
	ER	**91.4 (CR)**	**109.5 (CR)**	**691.4 (VHR)**	**310.2 (HR)**	**688.5 (VHR)**	**1641.0 (VHR)**	**29.1**
	ICF	**2.1 (MC)**	**1.5 (MC)**	**12.9 (VHC)**	**1.3 (MC)**	0.1	**18.1 (VHC)**	-
	RAC_m_	**35.5 (HR)**	0.0	1.5	**30.9 (HR)**	0.0	**61.4 (VHR)**	-
	ERF	**0.5 (MR)**	0.0	0.0	0.4	0.0	**1.6 (HR)**	-
S4	I_geo_	**2.9 (M-HC)**	−1.2	**2.5 (M-HC)**	0.2	**1.6 (MC)**	**3.9 (HC)**	**2.3 (M-HC)**
	ER	**337.4 (VHR)**	1.3	**41.3 (MR)**	8.7	22.0	22.9	**301.0 (HR)**
	ICF	**1.6 (MC)**	**1.2 (MC)**	**12.7 (VHC)**	**1.2 (MC)**	0.1	**16.0 (VHC)**	-
	RAC_m_	**25.0 (MR)**	0.0	1.2	**27.0 (MR)**	1.2	**55.8 (VHR)**	-
	ERF	0.3	0.0	0.0	0.4	0.0	**1.3 (HR)**	-
S5	I_geo_	**2.5 (M-HC)**	−1.4	**2.2 (M-HC)**	0.1	**1.4 (MC)**	**3.8 (HC)**	**3.5 (HC)**
	ER	**251.4 (HR)**	1.1	34.0	7.9	19.3	20.7	**684.4 (VHR)**
	ICF	**3.1 (CC)**	**1.5 (MC)**	**17.4 (VHC)**	**1.3 (MC)**	0.2	**28.6 (VHC)**	-
	RAC_m_	**37.7 (HR)**	2.0	3.8	**40.8 (HR)**	0.0	**68.7 (VHR)**	-
	ERF	**0.6 (MR)**	0.0	0.0	**0.7 (MR)**	0.0	**2.2 (HR)**	-
S6	I_geo_	**3.3 (HC)**	−1.1	**2.9 (M-HC)**	0.8	**2.0 (MC)**	**4.2 (HC)**	**3.5 (HC)**
	ER	**431.5 (VHR)**	1.4	**54.6 (MR)**	13.3	29.6	27.1	**692.7 (VHR)**
	ICF	**1.8 (MC)**	1.0	**12.8 (VHC)**	**1.1 (MC)**	0.1	**14.4 (VHC)**	-
	RAC_m_	**25.3 (MR)**	0.0	0.5	**34.8 (HR)**	0.0	**55.9 (VHR)**	-
	ERF	0.3	0.0	0.0	**0.5 (MR)**	0.0	**1.3 (HR)**	-
S7	I_geo_	**3.3 (HC)**	−1.1	**2.9 (M-HC)**	0.8	**2.0 (MC)**	**4.2 (HC)**	**3.4 (HC)**
	ER	**435.7 (VHR)**	1.4	**56.1 (MR)**	12.9	29.4	27.2	**627.2 (VHR)**
	ICF	**2.2 (MC)**	**1.2 (MC)**	**13.9 (VHC)**	**1.2 (MC)**	0.2	**16.7 (VHC)**	-
	RAC_m_	**23.4 (MR)**	2.4	1.4	**35.9 (HR)**	0.0	**55.0 (VHR)**	-
	ERF	0.3	0.0	0.0	**0.6 (MR)**	0.0	**1.2 (HR)**	-

**Reference**

In the original publication [1], there was a mistake in citation number 15. The incorrect date and act item. was used. There should be “Regulation of the Minister of Environment of 6 February 2015 on municipal sewage sludge J. L. 2015, Item. 257” instead of “Regulation of the Minister of Environment of 13th July 2010 on municipal sewage sludge J. L. 2010, No. 137, item. 924”. 

The corrected citation appears below.

[15] Regulation of the Minister of Environment of 6 February 2015 on the Municipal Sewage Sludge (J. L. 2015, Item. 257). Available online: https://isap.sejm.gov.pl/isap.nsf/DocDetails.xsp?id=wdu20150000257 (accessed on 29 July 2019).

The authors apologize for any inconvenience caused and state that the scientific conclusions are unaffected. The original publication has also been updated.
